# Development of liquid biopsy in detection and screening of pancreatic cancer

**DOI:** 10.3389/fonc.2024.1415260

**Published:** 2024-06-03

**Authors:** Xiangcheng Chen, Xinyi Hu, Tiancai Liu

**Affiliations:** ^1^ Key Laboratory of Antibody Engineering of Guangdong Higher Education Institutes, School of Laboratory Medicine and Biotechnology, Southern Medical University, Guangzhou, Guangdong, China; ^2^ School of The First Clinical Medical Sciences, Wenzhou Medical University, Wenzhou, Zhejiang, China

**Keywords:** liquid biopsy, pancreatic cancer, early detection, circulating tumor cells, circulating tumor DNA, extracellular vesicles, circulating tumor RNA

## Abstract

Pancreatic cancer is a highly lethal malignant tumor, which has the characteristics of occult onset, low early diagnosis rate, rapid development and poor prognosis. The reason for the high mortality is partly that pancreatic cancer is usually found in the late stage and missed the best opportunity for surgical resection. As a promising detection technology, liquid biopsy has the advantages of non-invasive, real-time and repeatable. In recent years, the continuous development of liquid biopsy has provided a new way for the detection and screening of pancreatic cancer. The update of biomarkers and detection tools has promoted the development of liquid biopsy. Circulating tumor cells (CTCs), circulating tumor DNA (ctDNA), circulating tumor RNA (ctRNA) and extracellular vesicles (EVs) provide many biomarkers for liquid biopsy of pancreatic cancer, and screening tools around them have also been developed. This review aims to report the application of liquid biopsy technology in the detection of pancreatic cancer patients, mainly introduces the biomarkers and some newly developed tools and platforms. We have also considered whether liquid biopsy technology can replace traditional tissue biopsy and the challenges it faces.

## 1 Introduction

Pancreatic cancer is a highly lethal malignant tumor. In 2024, it is estimated that new cases of pancreatic cancer in the United States will reach 66440, and deaths will reach 51750 (1). Although the 5-year relative survival rate of pancreatic cancer has greatly improved since 1975, it remains the lowest among all cancers, with only 13% (1). Pancreatic cancer has a high lifetime mortality to morbidity risk ratio and is expected to become the second leading cause of cancer death in the United States by 2026 (2). It is worth noting that the lifetime risk of pancreatic cancer continues to increase with the increase of the Human Development Index (HDI) level (2). Pancreatic cancer can be divided into resectable, borderline resectable, locally advanced or metastatic diseases (3). Surgical resection is a very effective treatment option at present (3, 4). The 5-year survival rate of patients with surgical resection was significantly higher than the actual 5-year survival rate of patients with pancreatic cancer at all stages. (4). However, many patients are in unresectable cancer at the time of diagnosis, and effective treatment methods are limited to chemotherapy, leading to poor prognosis of pancreatic cancer (3). Approximately 80% -85% of pancreatic ductal adenocarcinoma (PDAC) patients have locally advanced or distant metastatic diseases, only 15–20% of patients being diagnosed at the early stage of the disease (3).

As a promising detection technology, liquid biopsy has the advantages of non-invasive, real-time and repeatable. In 1994, it was first reported that mutant Kristen rat sarcoma (*KRAS*) sequences were detected in plasma circulating tumor DNA (ctDNA) of pancreatic cancer patients by PCR with allele specific primers, which means that liquid biopsy is promising for cancer detection (5). In the past decade, non-invasive methods of cancer diagnosis and monitoring, such as liquid biopsy, have gradually replaced invasive techniques such as tissue biopsy. Circulating tumor cells (CTCs), ctDNA, extracellular vesicles (EVs) and circulating tumor RNA (ctRNA) are the main targets of liquid biopsy. This review focuses on the latest research progress of liquid biopsy in the detection and screening of patients with pancreatic cancer, and evaluates its prospects and challenges.

## 2 Traditional detection methods for pancreatic cancer

Medical imaging plays an important role in early screening of pancreatic cancer. Computed tomography (CT) scanning, magnetic resonance imaging (MRI) and endoscopic ultrasonography (EUS) have been used to screen pancreatic cancer in high-risk groups with genetic syndrome or family history. Some scholars suggest using CT or MRI as a first-line investigation for suspected cancer patients, as this cross-sectional imaging modality is considered the gold standard for detecting primary lesions and distant metastases (6). EUS has become a powerful diagnostic modality that can provide high-resolution images of the pancreas, with quality far superior to other imaging techniques (7). In the past few years, endoscopic ultrasound-guided fine-needle aspiration (EUS-FNA) has been considered the most advanced and accurate diagnostic technique (7). A major advantage of EUS-FNA is its high sensitivity and specificity. A meta-analysis showed that a pooled sensitivity of EUS-FNA in diagnosing the correct etiology of pancreatic solid masses was 86.8%, and a pooled specificity of EUS-FNA was 95.8% (7). The overall complication rate of EUS-FNA is very low, and the safety of EUS-FNA is high. In 2011, Wang et al. analyzed 51 articles and found that the specific incidence of EUS-FNA was 0.98%, mostly pancreatitis and postoperative pain, and the mortality rate was only 0.02% (8). However, compared with non-invasive liquid biopsy technology, puncture will inevitably cause damage, and there is a risk of cancer cell bleeding and spread.

There is no effective serum tumor marker for early detection of pancreatic cancer in serological examination (6). Carbohydrate antigen 19–9 (CA19–9) is a recognized biomarker of pancreatic cancer, but the detection of CA19–9 often appears false positive and false negative. Experiments have shown that liver diseases, lung diseases, gynecological diseases, endocrine diseases, and spleen diseases can all lead to an increase in CA19–9, especially in cases of biliary obstruction (9). Furthermore, there is still a problem of insufficient secretion of CA19–9. As early as 1987, a study showed that Lewis antigen negative patients were unable to express CA19–9 in tumor tissue and had lower levels of CA19–9 in serum (10). As an aggressive subgroup with special clinical and molecular characteristics, Lewis negative pancreatic cancer accounts for about 5–10% of individuals (11). These factors have reduced the sensitivity and specificity of CA19–9 in the early detection of pancreatic cancer. An experiment proved that the sensitivity and specificity of CA19–9 level reached 60% and 99% within 0 to 6 months before diagnosis, and the sensitivity and specificity of cases diagnosed with early disease reached 50% and 99% (12). It can also effectively distinguish resectable pancreatic cancer from chronic pancreatitis and non-cancerous cysts, with specificity of 99% and sensitivity of 46% and 30%, respectively (12). In addition to CA19–9, other serological markers, such as carcinoembryonic antigen (CEA), carbohydrate antigen 125 (CA125) and carbohydrate antigen 242 (CA242), are also used for the detection of pancreatic cancer.

## 3 Liquid biopsy

### 3.1 Circulating tumor cells

CTCs are cells shed into the bloodstream from primary tumors and metastatic deposits (13). The presence of CTCs usually represents the invasion and metastasis of primary tumors, which is of great significance for the detection of pancreatic cancer (13). Accurately isolating and detecting CTC from a large number of blood cells is a major challenge, as their abundance in patient blood is extremely low. Current methods for CTC enrichment are either based on physical properties, such as size, density, or dielectrophoretic mobility (14). Or based on biological characteristics, CTCs are separated by immunoaffinity enrichment, which is divided into positive selection and negative selection (14). CTCs have many cell surface markers. The most common cell surface marker is epithelial cell adhesion molecule (EpCAM). It is a calcium dependent transmembrane glycoprotein that mediates epithelial cell adhesion and was detected by the CellSearch^®^ platform (14). However, biomarkers including EpCAM are significantly downregulated due to EMT in CTCs, which affects the detection rate of EpCAM positive CTCs (15). It suggests that relying solely on the detection of EpCAM-positive CTCs may not accurately reflect the full extent of the circulating tumor cell population (15). In addition to EpCAM, E-cadherin, vimentin and twist protein are also often used as molecular marker proteins to identify CTCs in pancreatic cancer.

As early as 20 years ago, two scholars mentioned in their articles that tumor cells can leave the primary lesion in the early stage of development (16). There is experimental evidence that CTCs can even spread in the early stages of tumor evolution. Rhim et al. detected circulating pancreatic cells in the blood of pancreatic intraepithelial neoplasia (PanIN) stage mice and found that some epithelial cells had undergone epithelial-to-mesenchymal transition (EMT) and entered the circulatory system (17). Later, Rhim et al. conducted experiments on PDAC patients, control population and patients with precancerous cystic disease. The detection rates were 73% (8/11), 0 (0/19) and 40% (8/21) respectively (18). CTCs could be detected in the blood of patients with precancerous cystic disease, suggesting that CTCs could be used for screening human pancreatic cancer.

A meta-analysis summarized dozens of literature between 2005 and 2020, and found a significant difference in the detection rate of CTCS in pancreatic cancer, with a total detection rate of 65%, but with very high specificity approaching 100% (19). The significant difference in the detection rate of CTC has raised doubts among many scientists about its diagnostic value. The currently widely used selection system is the CellSearch^®^ platform, which is also the only platform approved by the Food and Drug Administration (FDA) for detecting and separating CTCs. However, the CTC detection rate of the CellSearch^®^ system in pancreatic cancer is as low as 26% (19). Stoecklein et al. improved the sensitivity of CellSearch^®^ system detection by using diagnostic leukapheresis (DLA), significantly improving the detection rate of CTC in M0 and M1 patients with pancreatic cancer, with 44% and 74% respectively, resulting in a 60 fold increase in CTCs count (20).

### 3.2 Circulating tumor DNA

In the body fluid environment, a type of free DNA fragments that can be detected are called circulating free DNA(cfDNA). The cfDNA derived from tumor cells is called ctDNA. The genetic information carried by ctDNA is consistent with the tumor cells it is located in (21). DNA released from different tumor sites can provide a complete image of the tumor genome, which is considered to be more representative of heterogeneous cancer cells than genetic information obtained from single site tissue biopsy (21).

ctDNA fragment size and ctDNA level have potential to be used to identify patients with pancreatic cancer. In one study, the plasma of 61 patients with advanced pancreatic cancer and 28 healthy volunteers. The ctDNA fragment length of healthy control samples was longer than that of patient samples, and the ctDNA level of healthy control samples was significantly lower than that of patient samples (22). In the current research situation, it is known that the high mutation frequency of pancreatic cancer driving genes, such as *KRAS*. *KRAS* mutations were detected in plasma ctDNA of patients with pancreatic cancer as early as 1994 (5), and now *KRAS* mutations are widely used in diagnosis and monitoring. Combined detection of *KRAS* mutations and four protein markers increased sensitivity to 64% and specificity to 99.5% (23). It is worth noting that methylation analysis has been continuously developed in recent years. Epigenetic reprogramming, such as DNA methylation, occurs in the early stage of tumorigenesis and has potential as a target for early detection. In a 2024 experiment, comparative methylation analysis identified 9 differentially methylated loci in pancreatic exocrine DNA (24). Research shows that plasma ctDNA methylation has great potential to distinguish chronic pancreatitis from cancer pancreatic cancer (24). In a meta-analysis that assessed the utility of ctDNA for diagnosing cancer, it was found that liquid biopsy based on ctDNA had a sensitivity of 70% and a specificity of 86% (25). Low sensitivity has become the biggest obstacle to the detection of pancreatic cancer, so ctDNA detection is not suitable for large-scale screening. **Table 1** summarizes the basic information of some experiments with ctDNA detection to diagnose pancreatic cancer since 2023.

**Table 1:** Relevant experiments of cell-free nucleic acid in pancreatic cancer detection since 2023.

Target	Technology	Experiment objective	AUC	Sample source	Ref.
DNA methylation	Targeted ultra-deep NGS	Detection of PDAC	0.95	Serum	(26)
ctDNA	whole-genome sequencing	Distinguishing between normal and pancreatic cancer	0.66	Pancreatic juice	(27)
ctDNA	next-generation sequencing	Distinguish between early PDAC and normal	/	Pancreatic juice and serum	(28)
DNA methylation	next-generation sequencing	Distinguish between pancreato-biliary cancer and pancreatitis	0.88	Serum	(29)
DNA methylation	next-generation sequencing	Distinguishing between normal and pancreatic cancer	1.00	Serum	(30)
miRNA	RT-qPCR	Distinguishing malignant from benign disease	0.75	Bile	(31)
miRNA	RT-qPCR	Distinguishing PDAC from cholangiocarcinoma	0.81	Bile	(31)
lncRNA	PEViA-UC and PEViA-IP	Detection of PDAC	0.78	Serum	(32)
circRNA	RT-qPCR	Detection of PDAC	0.85	Serum	(33)
circRNA	RT-qPCR	Detection of early PDAC	0.83	Serum	(33)
miRNA	CRISPR-Cas12a powered hybrid nanoparticle	Detection of pancreatic cancer	0.94	Serum	(34)
mRNA	RT-qPCR	Identifying cystic precursor tumors	0.92	Pancreatic cyst fluid	(35)
miRNA	qPCR	Distinguishing between normal and pancreatic cancer	0.93	Serum	(36)
miRNA	ddPCR	Detection of PDAC	0.87	Serum	(37)
human satellite II RNA	ddPCR	Detection of PDAC	0.87	Serum	(37)

ctDNA is mixed with a large amount of DNA from non-cancer cells, so it is crucial to efficiently and accurately detect ctDNA. Currently, the technology available for ctDNA analysis is based on PCR or sequencing technology (38). At present, there are multiple commercial platforms available for detecting ctDNA from liquid biopsy, and research has been conducted on different *KRAS* ctDNA hotspot mutation detection platforms for in-depth evaluation (39). There are also studies evaluating the consistency of ctDNA mutation detection between the two most commonly used BEAMing and droplet digital PCR (ddPCR) (40). This comparison highlights the strong consistency between BEAMing and ddPCR, thus suggesting sufficient reproducibility for clinical applications.

### 3.3 Circulating tumor RNA

Like the definition of ctDNA, circulating cell-free RNA (cfRNA) from cancer cells is called ctRNA. Due to the protection of cell membrane-like structures and RNA binding proteins, as well as their own specific structure, they have a certain degree of stability. cfRNA includes many RNA types, such as microRNA (miRNA), long non-coding RNA (lncRNA), circular RNA (circRNA), small nuclear RNA (snRNA), small nucleolar RNA (snoRNA), and so on. Since 2023, the research of RNA in the detection of pancreatic cancer has attracted extensive attention from scientists all over the world, and the research has gradually become diversified. **Table 1** summarizes the basic information of some experiments related to this aspect since 2023. Interestingly, current experiments have different targets, methods, and sample sources. However, miRNA remains a hot topic in experimental research, reverse transcription-quantitative PCR (RT-qPCR) is the most popular technology today, and serum is the most commonly used sample source, with at least half of the experiments related to them.

Although cfRNAs include many RNA types, miRNAs are currently the focus of research and have received much attention. Shi et al. identified mir-1246, mir-205–5p, and mir-191–5p as serum biomarkers to identify pancreatic cancer (41). Moreover, the elevated levels of these miRNAs serve as significant indicators of the advanced stage of the disease, enabling the distinction between pancreatitis and pancreatic cancer with a precision of 91.5% (41). Prado et al. have established a distinctive miRNA signature in bile, which serves as an effective tool to differentiate between malignant and benign pancreaticobiliary diseases (31). Among these, mir-148a-3p has been particularly identified as a unique marker and its efficacy in distinguishing the two conditions has been validated (31). Furthermore, a dual miRNA signature encompassing mir-125b-5p and mir-194–5p has been formulated, enabling the precise differentiation of PDAC from cholangiocarcinoma (31).

### 3.4 Extracellular vesicles

EVs are lipid bilayer particles secreted by cells into the extracellular space. EVs can carry cellular components such as DNA, RNA, proteins, lipids, amino acids and metabolites, reflecting the state of the cell. Now, according to their biogenesis, content and secretion pathway, EVs can be divided into two major categories: exosomes and microvesicles. A comprehensive description of the nomenclature, collection and pre-processing, separation and concentration, characterization, functional studies and some general considerations of EVs was provided in the Minimum information for studies of extracellar vessels (MISEV2018). In order to provide researchers with the latest snapshot of available methods, MISEV2018 has been recently modified. The release of MISEV2023 aims to encourage more researchers to invest in the research field of EVs biomarkers and the therapeutic potential of EVs (42).

Compared to the previously mentioned ctDNA and CTC, EVs have unique advantages, with the highest sensitivity and specificity (25). Similar to ctDNA, *KRAS* mutations can also be detected in exosomal DNA with better sensitivity and specificity. Allenson et al. conducted experiments in age matched control groups, limited, locally advanced and metastatic PDAC patients, and found *KRAS* mutations in 7.4%, 66.7%, 80% and 85% of exosomal DNA, respectively (43). The experiment also compared exosomal DNA with ctDNA, and PDAC patients showed a higher proportion of detectable *KRAS* mutations in exosomal DNA (43). In addition, the methylation of exosomal DNA has also been confirmed to be different, which has the potential to distinguish chronic pancreatitis from pancreatic cancer (24).

EVs not only contain DNA but also RNA, which can also serve as biomarkers for tumor detection. Among them, exosomal miRNA is currently a hot research topic. The expression of miRNA-191, miRNA-21 and miRNA-451a in EVs of pancreatic cancer and IPMN patients was significantly up-regulated compared with the control group (44). Experiments have proved that the levels of these three exosomal miRNAs can be used as early diagnosis and progression markers of pancreatic cancer and IPMN, and are considered more useful than circulating miRNAs (44). Makler et al. analyzed the exosomal miRNAs in cell culture medium and detected the expression of miRNAs in pancreatic cancer (45). Seven mature miRNAs showed statistical significance, and these identified miRNAs have the potential for early detection of PDAC (45).

In 2015, a study identified a cell surface protein polysaccharide called glycan-1 (GPC1), which is particularly enriched in cancer cell-derived exosomes (46). The detection of GPC1-positive exosomes (GPC1-Exos) in the serum of pancreatic cancer patients has absolute specificity and sensitivity, which can distinguish healthy subjects and patients with a benign pancreatic disease from patients with early- and late-stage pancreatic cancer (46). Lewis et al. integrated direct exosomes and other extracellular vesicles onto an alternating current microarray chip to detect PDAC using biomarkers GPC-1 and CD63, with a sensitivity of 99% and specificity of 82% (47). In 2023, a graphene field-effect transistor (GFET) based biosensor was successfully developed for detecting PDAC through GPC-1 expression within 45 minutes (48). The GFET biosensor array can accurately distinguish between PDAC patients and healthy controls, while also being able to detect early stages of cancer, including stages 1 and 2 (48). Yang et al. identified the signature of five markers for PDAC detection, and one of the best markers, GPC1 alone, had 82% sensitivity and only 52% specificity (49). However, the combined detection of five marker signals showed 86% sensitivity and 81% specificity (49). **Table 2** summarizes the relevant experiments of the citations that have appeared.

**Table 2:** Summary of some researches on the use of EVs detection for pancreatic cancer diagnosis.

Target	Method	Technology	Experiment objective	AUC	Ref.
exoDNA	*KRAS*	ddPCR	Compare exoDNA to ctDNA in liquid biopsies	/	(50)
exoDNA	Pancreas-­specific methylation	Whole genome bisulfite sequencing	Distinguish between early PDAC and normal	0.95	(24)
miRNA	ExmiR-191	RT-qPCR	Diagnosing PC	0.79	(44)
miRNA	ExmiR-21	RT-qPCR	Diagnosing PC	0.83	(44)
miRNA	ExmiR-451a	RT-qPCR	Diagnosing PC	0.76	(44)
Cell surface markers	GPC1	Flow cytometry	Differentiate between pancreatic cancer, benign pancreatic disease and normal	1.00	(46)
Cell surface markers	GPC1 and CD63	Integrated analysis of exosomal protein on alternating current electrokinetic chips	Distinguish between PDAC and normal	1.00	(47)
Cell surface markers	GPC1	Graphene Sensor Arrays	Distinguish between PDAC and normal	/	(48)

## 4 Discussion

Liquid biopsy technology has significant advantages over tissue biopsy due to its non-invasive, real-time, and high reproducibility characteristics. The biggest obstacle of liquid biopsy is that the sensitivity and specificity of early detection are not high enough. None of the various biomarkers can provide the required high sensitivity and specificity for early detection of pancreatic cancer. However, the current multi-component combination detection can to some extent improve sensitivity and specificity, and improve diagnostic accuracy. The development of new detection tools and the introduction of new technologies are pushing liquid biopsy to a new climax. Entering clinical practice, liquid biopsy still needs to be studied in practical clinical applications, and the technology needs to be standardized and rationalized. In conclusion, liquid biopsy technology has a good application prospect in the detection of pancreatic cancer, but more researchers need to invest in further in-depth research.

## Author contributions

XC: Conceptualization, Methodology, Writing – original draft. XH: Conceptualization, Methodology, Writing – review & editing. TL: Methodology, Supervision, Writing – review & editing.
